# Fibroblast growth factor receptor 2 promotes the proliferation, migration, and invasion of ectopic stromal cells via activation of extracellular-signal-regulated kinase signaling pathway in endometriosis

**DOI:** 10.1080/21655979.2022.2054207

**Published:** 2022-03-21

**Authors:** Yuan Xu, Feng Gao, Jingyong Zhang, Pingping Cai, Dongsheng Xu

**Affiliations:** a Traditional Chinese Medicine Department, Shandong Provincial Hospital Affiliated to Shandong First Medical University, Jinan, P.R. China; b Biomedical Isotope Research Center, School of Basic Medical Sciences, Shandong University, Jinan, P.R. China; c Vascular Surgery Department, Shandong Provincial Hospital Affiliated to Shandong First Medical University, Jinan, P.R. China; d Department of Kidney Transplantation, The Second Hospital, Cheeloo College of Medicine, Shandong University, P.R. China

**Keywords:** Endometriosis, ectopic stromal cell, FGFR2, ERK signaling

## Abstract

Endometriosis is defined as the presence of endometrial tissues with cancer-like features in extrauterine locations. Fibroblast growth factor receptor 2 (FGFR2) is a tyrosine kinase that is involved in cancer pathogenesis. This study aimed to determine the role of FGFR2 in endometriosis. A total of 29 pairs of ectopic and eutopic endometrial tissues were collected from women with endometriosis. Endometrial tissues from women with hysteromyomas were considered as normal controls. Primary ectopic stromal cells (ESCs) were isolated from the ectopic endometrium. The role of FGFR2 in ESCs was assessed using immunohistochemistry, polymerase chain reaction, cell counting kit-8 assay, EdU staining, flow cytometry, transwell assay, and western blotting. The following signaling pathways were detected using bioinformatic analysis and confirmed *in vitro*. By searching the GSE171154, GSE86543, and GSE77182 datasets, FGFR2 was identified as an upregulated overlapping gene in endometriosis. Compared to eutopic and normal endometria, FGFR2 was highly expressed in ectopic tissues. Transfection of primary ESCs with FGFR2 small interfering RNA (siRNA) repressed the viability and proliferation of cells and induced apoptosis. FGFR2 siRNA inhibited the migration, invasion, and transforming growth factor-β1-triggered epithelial-mesenchymal transition (EMT). Extracellular signal-regulated kinase (ERK) signaling was found to be a downstream signaling pathway for FGFR2. The ERK1/2 inhibitor PD98059 was found to reverse the promoting effects of FGFR2 on ESC proliferation and invasion. FGFR2 silencing effectively inhibited the growth, migration, invasion, and EMT of ESCs. The effects of FGFR2 on endometriosis might be mediated via the activation of ERK signaling.

## Graphical abstract

**Figure:**
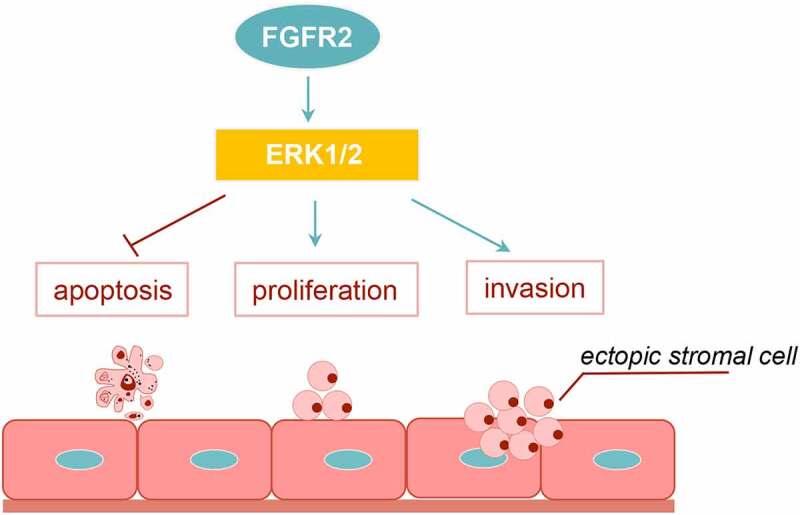

## Introduction

Endometriosis is a chronic inflammatory gynecological disease characterized by the presence of endometrial tissue in extrauterine locations. Endometriosis includes a wide spectrum of symptoms such as pelvic pain, irregular menstrual cycle, constipation, and abdominal bloating, which can significantly impact the quality of life [1,2]. In addition to these symptoms, endometriosis may lead to infertility [3]. Statistically, the incidence of endometriosis among reproductive-aged women ranges from 2–5%, and the prevalence of infertility ranges from 20–50% [4]. Presently, no single successful therapeutic option is available for endometriosis, making its treatment very challenging [5]. In the event of endometriosis, high proliferation of ectopic endometrial cells and endometrial metastasis, depending on invasive and migrating mechanisms, contribute substantially to the progression of endometriosis [6]. Therapeutic targets for inhibiting ectopic stromal cell (ESC) proliferation, migration, and invasion are considered to have potential for treating endometriosis [7,8].

Fibroblast growth factor receptor 2 (FGFR2) is a member of the FGFR family and is remarkably involved in human cancer pathogenesis [9]. FGFR2 plays a critical role in promoting carcinogenesis in various cancers, such as gastric cancer [10], cholangiocarcinoma [11], colorectal cancer [12], and endometrial cancer [13]. In normal endometrium, FGFR is highly expressed in the secretory phase compared to the proliferative phase, and its expression contributes to the tumourigenesis of endometrial carcinoma [14]. There is current knowledge regarding the role of FGFR2 in embryo development [15] and eyelid, skin, and bone formation [16,17]. However, there are no studies available that have reported the expression and effects of FGFR2 in endometriosis.

Extracellular signal-regulated kinases (ERKs), c-Jun amino-terminal kinases (JNKs), and p38 are members of larger family of mitogen-activated protein kinases (MAPK) [18]. ERKs are cytoplasmic serine and threonine kinases that can be activated rapidly by numerous extracellular and intracellular stimuli, such as viral infection [19], oxidative stress [20], cytokines [21], and the tumor microenvironment [^22–24^]. In addition, the ERK signaling pathway is known to be involved in the development of various human diseases, including neurodegenerative diseases [25], heart diseases [26], renal fibrosis [27], and cancer [^22–24^]. Regarding endometriosis, ERK signaling is activated by hormones and contributes to endometriosis lesion growth, inflammation, and ESC epithelial-mesenchymal transition (EMT) [28,29].

In the present study, differentially upregulated genes in endometriosis were analyzed from the GSE171154, GSE86543, and GSE77182 datasets, and FGFR2 was identified as an overlapping gene. The expression and functional effects of FGFR2 in endometriosis were also studied. The primary ESCs were isolated from endometriosis women who underwent laparoscopic surgery. FGFR2 expression in ESCs was silenced and overexpressed by transfection with specific small interfering RNAs (siRNAs) and overexpression plasmids, respectively. The growth, migration, invasion, and transforming growth factor-β1 (TGF-β1)-triggered EMT of ESCs were examined to evaluate the role of FGFR2 in endometriosis. In addition, the regulation of the FGFR2 and MAPK signaling pathways was investigated to reveal the underlying mechanisms.

## Materials and methods

### Sample collection

A total of 29 women (aged 26–53 years; median age of 41.6 ± 8.1 years) with ovarian endometriosis were enrolled in this study. Ectopic and paired eutopic endometrial tissues were collected during laparoscopic surgery between February 2020 and September 2021 at the Second Hospital, Cheeloo College of Medicine, Shandong University. All the enrolled women were diagnosed using histological examinations and diagnostic criteria, as previously reported [30]. None of the patients had received sex hormones or anti-endometriosis drugs for at least six months prior to the operation. In addition, 23 women (aged 33–55 years; median age of 46.5 ± 7.0 years) with hysteromyoma were enrolled. None of the enrolled patients were menopausal and all tissues were derived from the proliferative phase of the menstrual cycle. Endometrial tissues were collected during hysterectomy and used as positive controls. This present experimental study was approved by the Ethics Committee of the Second Hospital, Cheeloo College of Medicine, Shandong University (Ethic numbers: 2020–065). Written informed consent was obtained from all participants.

### Immunohistochemistry (IHC)

The collected endometrial tissues were washed twice with phosphate-buffered saline (PBS), embedded in paraffin, sectioned into 5 μm specimens, de-paraffinized, rehydrated, and blocked with 6% skim milk for 1 h at room temperature. The slices were incubated with a mouse monoclonal antibody for FGFR2 (1:100; LifeSpan BioSciences, USA) at 4°C overnight and a rat anti-mouse biotinylated secondary antibody (1:100; eBioscience™, USA) at room temperature for 1 h. VECTASTAIN® ABC kits (HRP) (Vector Laboratories, USA) were used to detect biotinylated targets at room temperature for 30 min. Diaminobenzidine (Vector Laboratories, USA) was used as the final chromogen according to the manufacturer’s protocol. The calculation of IHC score was examined by two histopathologists blinded to the protocol. The IHC scores are graded as follows: the staining intensity (0, no staining; 1, weak staining; 2, moderate staining; and 3, strong staining) and the percentage of positive staining (0, 0–5% positive area; 1, 6–25% positive area; 2, 26–50% positive area; 3, 51–75% positive area; and 4, 76–100% positive area) were recorded, and the total score was calculated by multiplying the two scores [31].

### Isolation of ESCs

The endometrial tissues collected from patients with ovarian endometriosis were washed twice with PBS, cut into approximately 5 mm^3^ pieces, and digested with 2.5 mg/ml type IV collagenase (Sigma-Aldrich, USA) for 1 h at 37°C and 15 U/ml deoxyribonuclease (Sangon Biotech, China) for 30 min. ESCs in the cell suspension were purified according to the following procedure: filtered through a 70 μm cell strainer, filtered through a 50 μm cell strainer, and washed twice with PBS. ESCs were cultured in DMEM/F12 medium (Procell, China) supplemented with 10% fetal bovine serum (FBS; Hyclone, USA), and maintained at 37°C with 5% CO_2_. ESCs at passage two were used for immunofluorescence staining for detection of vimentin and cytokeratin. ESCs with purity greater than 90% were used in subsequent studies.

Recombinant human TGF-β1 (Sangon Biotech, China) at concentrations of 0–10 ng/ml was used to treat ESCs for 24 h to induce EMT [32,33]. The ERK1/2 inhibitor PD98059 (MedChemExpress, USA) at a density of 30 μM was used to treat the cells for 24 h.

### Bioinformatic analysis

The GSE171154, GSE86543, and GSE77182 datasets from the Gene Expression Omnibus database (www.ncbi.nlm.nih.gov/geo) were used to analyze endometriosis-related genes. The GSE171154 dataset comprises endometrial specimens from four patients with endometriosis and four patients without endometriosis. The GSE86543 dataset comprises mRNA information from two endometrial cancer cell lines, Ishikawa and Hec1a. The GSE77182 dataset comprises five sets of pooled samples; these include ten endometriosis serum samples, ten control serum samples, five eutopic endometrial tissue samples, five ectopic endometrial tissue samples, and five control tissue samples. The upregulated genes in the endometriosis groups were analyzed using the “limma’ package in R language.

Kyoto Encyclopedia of Genes and Genomes (KEGG) and Wiki pathway enrichment analyzes were performed to determine the mechanism underlying FGFR2-mediated endometriosis using the ClusterProfiler package in R language. The STRING database was used to construct protein-protein interactive networks.

### Cell transfection

Human siRNAs specific for FGFR2 with the sequences 5’-GCCCAACA ATAGGACAGTGCT-3’ (si-1) and 5’-GGTCTTCTTAATCGCCTGTAT-3’ (si-2) were designed and purchased from GenePharma (Shanghai, China). Scrambled 5’-GTGCAAACCCGAGGCAACTAT-3’ was used as a negative control (si-NC). Full-length FGFR2 was amplified using polymerase chain reaction (PCR). The PCR products were inserted into the pcDNA3.1 plasmid (Invitrogen, USA) using T4DNA ligase (Thermo Scientific ®, USA) to construct the FGFR2 overexpression plasmid pcFGFR2. We then transfected ESCs in 6-well plates at 70–80% confluence with 20 μg/ml plasmids or 100 nM siRNAs using Lipofectamine 2000 (Invitrogen, USA). After incubation in serum-free culture medium for 12 h at 37°C, the cells were maintained in complete culture medium (with 10% FBS) for 48 h and collected for use in subsequent experiments.

### Quantitative reverse transcription PCR (qRT-PCR)

Total RNA from endometrial tissues and ESCs was extracted using a Total RNA Extraction Kit (Solarbio, China). Goldenstar RT6 cDNA Synthesis Mix (Tsingke Biotechnology, China) and 2× T5 Super PCR Mix (Basic) (Tsingke Biotechnology, China) were used for reverse transcription and quantitative PCR, respectively. Relative FGFR2 expression was calculated by normalizing to β-actin using the 2^−ΔΔCq^ method (22). The primer sequences used were as follows: FGFR2 forward 5’-CCTGGATGTTGTGGAGCGAT-3’, reverse 5’-CTGTTACCTGTCTCCGCAGG-3’; β-actin forward 5’-GATTCCTATGTGGGCGACGA-3’, reverse 5’-CACAGGACTCC ATGCCCAG-3’.

### Western blot

Proteins in endometrial tissues and ESCs were isolated using a radioimmunoprecipitation assay buffer (Solarbio, China). The protein concentration of the lysate was determined using a Bicinchoninic acid Protein Assay Kit (Solarbio, China), and 40 μg of protein from each sample were subjected to 10–12% SDS gels and transferred to PVDF membranes (Millipore, USA). After incubation with 5% nonfat milk for 1 h, the membranes were probed with the following primary antibodies at 4°C overnight: FGFR2 (1:2000; LifeSpan BioSciences, USA), E-cadherin (1:2000; Invitrogen, USA), N-cadherin (1:1000; Invitrogen, USA), vimentin (1:1000; Invitrogen, USA), ERK1 (phospho T202 + Y204) + ERK2 (phospho T185 + Y187) (1:1000; Abcam, USA), ERK1/2 (1:10,000; Abcam, USA), p38 (phospho T180) (1:1000; Abcam, USA), p38 (1:1000; Abcam, USA), JNK (phospho T183 + T183 + T221) (1:1000; Abcam, USA), JNK (1:1000; Abcam, USA), and β-actin (1:2000; Abcam, USA). After incubation with rat anti-mouse (1:1000; eBioscience™, USA) and goat anti-rabbit secondary antibodies (1:2000; Abcam, USA), immunoblots were developed using the ECL method.

### Cell counting kit-8 (CCK-8)

Post-transfection, 2 × 10^3^ ESCs were seeded in 96-well plates. The viability of ESCs after 24, 48 and 72 h of incubation was determined using the CCK-8 Cell Proliferation and Cytotoxicity Assay Kit (Solarbio, China). Briefly, 10 µl CCK-8 solution was added to the culture. After 4 h of incubation at 37°C, the optical density value was assessed using a microplate reader (Bio-Rad, USA) at 450 nm.

### EdU assay

Post-transfection, 4 × 10^3^ ESCs were seeded in 96-well plates. For EdU staining, 50 µM EdU reagent (Thermo Fisher Scientific, USA) in the culture medium was added to each well and incubated for 1 h at 37°C. DAPI (0.1 μg/ml) was used for nuclei staining. Staining results were observed using a fluorescence microscope (Leica, Germany).

### Flow cytometry

After transfection, 3 × 10^5^ ESCs were seeded in 6-well plates. For early and late apoptotic cell detection, an Annexin V-FITC/PI kit (Beyotime, China) was used according to the manufacturer’s protocol. Samples with at least 1 × 10^5^ ESCs were analyzed using flow cytometry (BD Biosciences, USA).

### Transwell assay

After transfection, the migration and invasion of ESCs were analyzed using a transwell system (Costar-Corning, USA) in the absence or presence of Matrigel pre-coating (BD Biosciences, USA). ESCs were starved in non-serum culture medium for 12 h, and 100 μl of the cell suspension (5 × 10^5^/ml) was added to the upper chamber. The lower chamber was filled with 600 μl of complete culture medium (with 10% FBS) as a chemoattractant. After 48 h, the ESCs in the lower chamber were stained with 0.1% crystal violet (Sangon Biotech, China) for 20 min. The images were captured under a microscope (Leica), and the number of stained cells was calculated

### Statistical analysis

Data are shown as the mean + standard deviation from at least three independent experiments. One- or two-way ANOVA was conducted using GraphPad Software 6.0 for multiple groups of comparisons. Statistical significance was set at *p* < 0.05.

## Results

### FGFR2 is highly expressed in endometriosis

**Figure 1.**
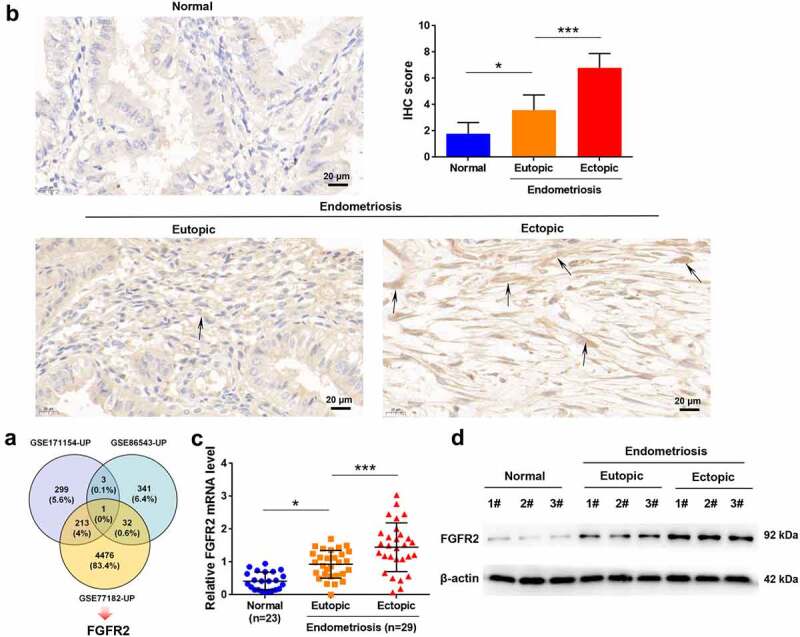
The expression of FGFR2 in endometriosis. (a) FGFR2 was identified as an overlapping gene from GSE171154, GSE86543, and GSE77182 datasets. (b) Expression of FGFR2 in normal control (n = 5) and endometriosis tissues (n = 5) was detected by immunohistochemistry (magnification 400×). Endometrial tissues from women with hysteromyomas were considered as normal controls. (c) mRNA level of FGFR2 in normal controls (n = 23) and endometriosis tissues (n = 29) was detected by qRT-PCR. (d) Protein level of FGFR2 in normal controls (n = 3) and endometriosis tissues (n = 3) was detected by western immunoblotting. *p < 0.05, ***p < 0.01 vs. the indicated group.

By analyzing the GSE171154, GSE86543, and GSE77182 datasets, FGFR2 was identified as an upregulated gene in endometriosis (Figure 1a). To validate the upregulation of FGFR2 in endometriosis, its expression was detected in endometrial tissues collected from patients with endometriosis and normal controls. In contrast with the normal tissues, both eutopic and ectopic endometria from patients with endometriosis had remarkably higher FGFR2 levels (Figure 1b). In addition, ectopic tissues showed higher FGFR2 levels than the paired eutopic tissues (*p* < 0.05). The high expression levels of FGFR2 in endometriosis tissues were also confirmed using qRT-PCR (*p* < 0.05; Figure 1c) and western blot analysis (Figure 1d). These data show that FGFR2 is highly expressed in endometriosis.

### FGFR2 silencing inhibits proliferation and induces apoptosis of ESCs

**Figure 2.**
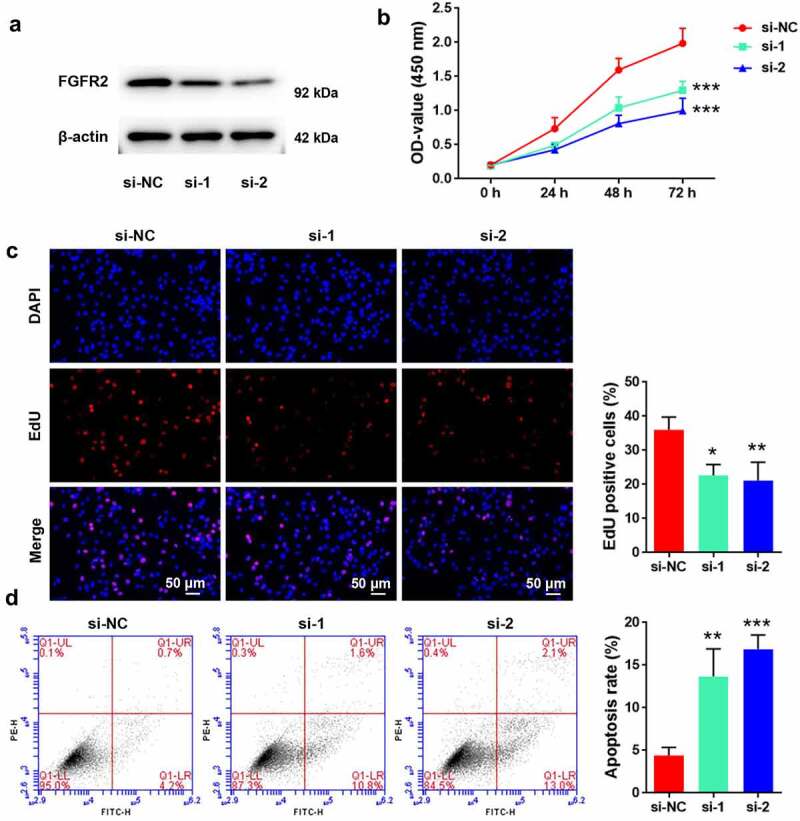
Effects of FGFR2 on ESC proliferation and apoptosis. (a) FGFR2 siRNAs (si-1 and si-2) or the negative control (si-NC) were transfected into primary ESCs which were isolated from human ectopic endometrium. Protein level of FGFR2 was detected by western blotting. (b) Cell viability, (c) proliferation, and (d) apoptosis were determined by CCK-8 assay, EdU staining, and flow cytometry, respectively. *p < 0.05, **p < 0.01, ***p < 0.01 vs. si-NC group.

To reveal the potential roles of FGFR2 in the development of endometriosis, its expression in primary ESCs isolated from human endometriosis tissues was silenced by siRNA transfection. Transfection of ESCs with FGFR2 siRNAs (si-1 and si-2) markedly reduced FGFR2 expression levels compared to transfection with si-NC (Figure 2a). Consistent with the si-NC group, cells transfected with FGFR2 siRNA showed significant loss in viability (*p* < 0.05; Figure 2b) and proliferation inhibition (*p* < 0.05; Figure 2c). Moreover, cells transfected with FGFR2 siRNA induced significant apoptosis-related death compared to those transfected with si-NC (*p* < 0.05; Figure 2d). These results suggest that FGFR2 silencing is effective in inhibiting ESC proliferation and inducing apoptosis.

### FGFR2 silencing inhibits migration, invasion, and TGF-β1-induced EMT of ESCs

**Figure 3.**
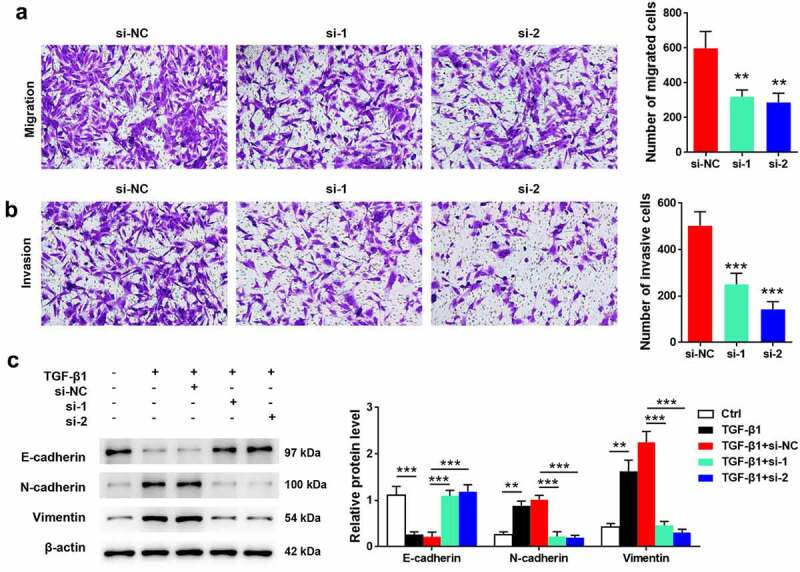
Effects of FGFR2 on ESC migration, invasion, and TGF-β1-induced EMT. FGFR2 siRNAs (si-1 and si-2) or the negative control (si-NC) were transfected into ESCs. Transwell assays were performed for detecting (a) cell migration and (b) invasion. (c) Cells were treated with 10 ng/ml TGF-β1 for 24 h. Expression of EMT-related proteins was detected by western blotting. **p < 0.01, ***p < 0.01 vs. si-NC group or the indicated group.

As detected using transwell assay, cells transfected with FGFR2 siRNAs had a lower number of migrated and invasive cells than those transfected with si-NC (*p* < 0.05; Figure 3a**, 3B**). Additionally, the effects of FGFR2 silencing on TGF-β1-induced EMT were determined. Treatment of cells with TGF-β1 induced significant downregulation of E-cadherin and upregulation of N-cadherin and vimentin (*p* < 0.05; Figure 3c). FGFR2 siRNA reversed the effects of TGF-β1 on the protein expression of E-cadherin, N-cadherin, and vimentin relative to those in the si-NC group (*p* < 0.05). These results suggest that FGFR2 silencing inhibited the migration, invasion, and TGF-β1-induced EMT in ESCs.

### FGFR2 silencing inhibits ERK signaling pathway in ESCs

**Figure 4.**
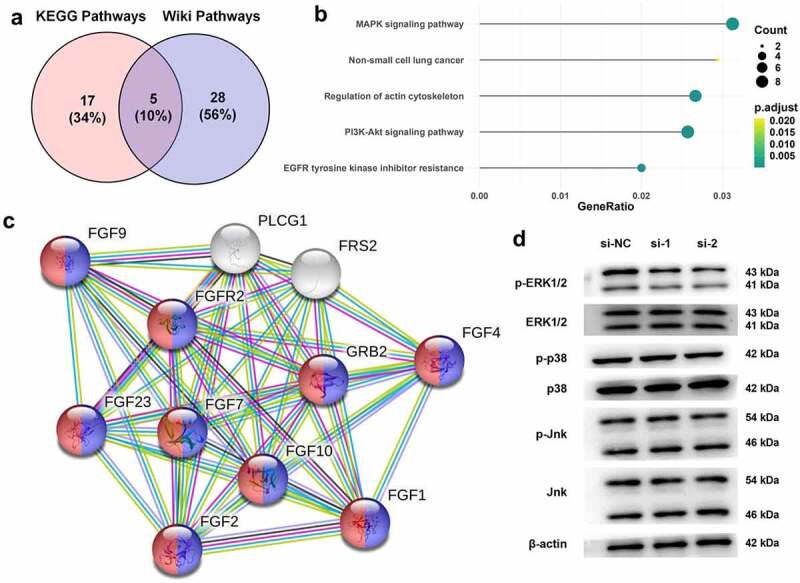
Regulation between FGFR2 and ERK signaling pathway in ESCs. (a) KEGG and Wiki pathway enrichment analyzes were performed for analyzing endometriosis-related genes. (b) The overlapped five signaling pathways were shown in bubble diagram. (c) The interaction between FGFR2 and MAPK signaling pathway proteins were constructed by STRING database. (d) Expression of ERK1/2, p38, and Jnk in ESCs following the transfection of FGFR2 siRNAs (si-1 and si-2) or si-NC was detected by western blotting.

To further reveal the mechanism underlying the anti-endometriosis effects of FGFR2 silencing, KEGG and Wiki pathway enrichment analyzes were performed (Figure 4a). Five biological processes and pathways were screened, namely the MAPK signaling pathways, non-small cell lung cancer, regulation of actin cytoskeleton, PI3K-Akt signaling pathway, and EGFR tyrosine kinase inhibitor resistance. Among these, the MAPK signaling pathway with the highest enrichment score was selected for subsequent studies (Figure 4b). The interaction between MAPK signaling pathway proteins and FGFR2 were constructed using the STRING database (Figure 4c). The regulation of FGFR2 in the MAPK signaling pathway in ESCs was confirmed. Immunoblotting results in Figure 4d show that FGFR2 siRNAs remarkably downregulated the expression of the phosphorylated form of ERK1/2 but had no effect on the total levels of ERK1/2. FGFR2 siRNAs also had no substantial effect on the expression of p38 and JNK. It seems that silencing FGFR2 deactivates the ERK signaling pathway, but not the p38 and JNK signaling pathways in ESCs.

### FGFR2 promotes ESC proliferation and invasion via activation of ERK signaling

**Figure 5.**
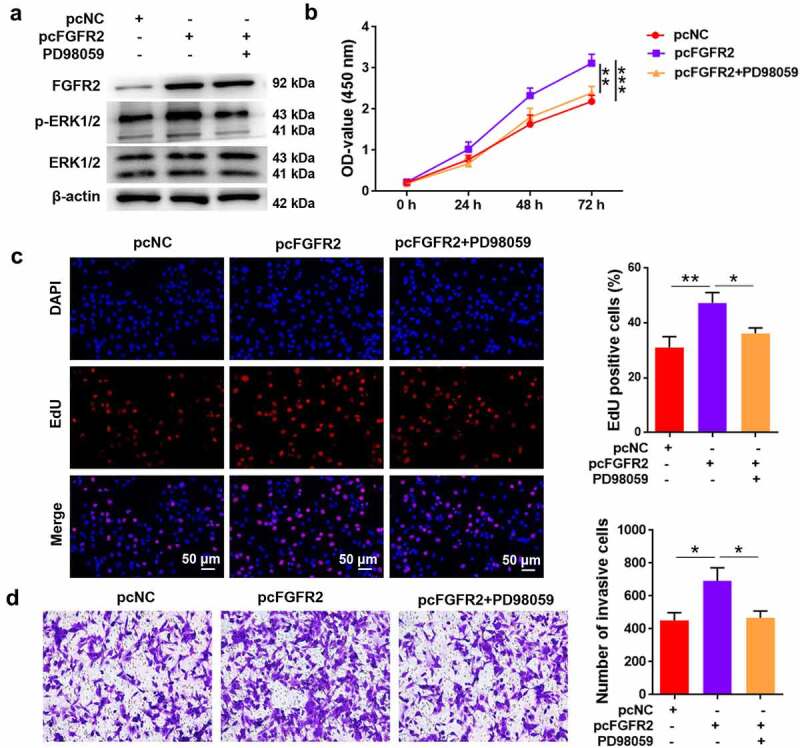
Involvement ERK signaling in FGFR2-mediated proliferation and invasion in ESCs. ESCs transfected with FGFR2 overexpressing plasmid (pcFGFR2) or pcNC were treated with 30 μM PD98059 for 24 h. (a) Expression of FGFR2 and ERK1/2 was detected by western blotting. (b) Cell viability, (c) proliferation, and (d) invasion were detected by CCK-8 assay, EdU staining, and transwell assay, respectively. *p < 0.05, **p < 0.01, ***p < 0.01 vs. the indicated group.

Furthermore, the ERK1/2 inhibitor PD98059 was used to treat ESCs to reveal the involvement of ERK signaling in FGFR2-mediated endometriosis. Transfection of ESCs with FGFR2 overexpressing plasmid (pcFGFR2) induced remarkable upregulation of FGFR2 and the phosphorylated form of ERK1/2 (Figure 5a). The activation of ERK1/2 induced by pcFGFR2 was reversed by PD98059 treatment. Transfection of ESCs with pcFGFR2 induced a significant increase in cell viability and proliferation compared to pcNC (*p* < 0.05; Figure 5b**, 5C**). However, treatment of ESCs with PD98059 significantly abolished the effects of pcFGFR2 on cell viability and proliferation (*p* < 0.05). Similarly, ESCs transfected with pcFGFR2 showed significant invasion, which was inhibited by PD98059 treatment (*p* < 0.05; Figure 5d). Collectively, these results show that FGFR2 participates in ESC proliferation and invasion and possibly via the activation of ERK signaling.

## Discussion

The presence of ESCs outside the uterine cavity is one of the key features of endometriosis [6]. In this study, primary ESCs were isolated from women with endometriosis who underwent laparoscopic surgery. FGFR2 was found to be highly expressed in ectopic endometrial tissues. FGFR2 silencing in ESCs significantly inhibited cell viability and proliferation, which was accompanied by an increase in apoptosis. In addition, FGFR2 silencing inhibited the migration, invasion, and TGF-β1-induced EMT in ESCs. ERK signaling, in contrast to p38 and JNK signaling, was found to be a downstream signaling pathway contributing to FGFR2 and exerting its effects in endometriosis.

FGFR2 is a well-known tyrosine kinase expressed in two distinct isoforms, FGFR2-IIIb and FGFR2-IIIc, depending on tissue-specific alternative splicing [34]. In general, FGFR2-IIIb is expressed in epithelial cells, and FGFR2-IIIc is expressed primarily in mesenchymal cells [35]. Thus, the isoforms of FGFR2 have been considered to have a significant impact on epithelial–stromal interactions in human cancers [36]. In addition, FGFR2 is highly expressed in various solid tumors, including gastric and endometrial cancers [10,13], indicating that FGFR2 is a potential biomarker of human cancers. Although endometriosis is considered to have cancer-like features, such as uncontrolled cell proliferation, local invasion, and apoptosis resistance [37], less attention has been paid to the expression of FGFR2 in endometriosis. In the current study, FGFR2 was screened from three datasets (GSE171154, GSE86543, and GSE77182) as an upregulated overlapping gene in endometriosis. Further studies confirmed that FGFR2 was highly expressed in the ectopic endometrium of patients with endometriosis than in eutopic and normal endometria. These data indicated an elevated level of FGFR2 in endometriosis. In addition, FGFR has been previously reported to be highly expressed in the secretory phase compared to the proliferative phase in normal endometrium, suggesting its contribution in the tumourigenesis of endometrial carcinoma [14]. Considering irregular menstrual cycle is also a risk factor for endometriosis [38], the expression between endometriotic endometria in the secretory and proliferative phases should be further revealed.

Considering that FGFR2 is highly expressed in endometriosis, the fundamental involvement of FGFR2 in disease development was studied. By performing *in vitro* studies on primary ESCs collected from women with endometriosis, FGFR2 was found to be effective in promoting ESC proliferation, migration, and invasion and inhibiting apoptosis. In addition, FGFR2 has remarkable promoting effects on TGF-β1-triggered EMT process, as evidenced by the dysregulated expression of E-cadherin, N-cadherin, and vimentin. The role of FGFR2 in ESCs is similar to its role in other cancer cell lines, including gastric [39], thyroid [36], and colorectal cancer cell lines [40]. These data demonstrate that FGFR2 is a potential therapeutic target for the treatment of endometriosis. However, before FGFR2 targeting can be used in the clinical setting, several *in vivo* and *in vitro* studies are required to confirm its benefits and side effects.

Various kinase signaling pathways are activated in endometriosis, including the MAPK signaling pathways [41]. Activated MAPK signaling in endometriosis plays a decisive role in mediating endometriosis lesion growth, inflammation, and the EMT process of ESCs [28,29]. Inhibition of MAPK by sorafenib or luteolin can control endometriosis progression [42,43]. In addition, MAPK signaling has been recognized as the main downstream signaling pathway mediated by FGFR2 [44]. In this study, KEGG and Wiki pathway enrichment analyzes were performed to analyze endometriosis-related genes, and the MAPK signaling pathway was identified as the most involved signaling pathway in endometriosis development. Further studies demonstrated that ERK signaling, rather than p38 and JNK signaling, is a downstream signaling pathway responsible for FGFR2 effects in endometriosis. Using an ERK1/2 inhibitor (PD98059), the promoting effects of FGFR2 on ESC proliferation and invasion were reversed. These findings suggest that ERK is one of the main signaling pathways involved in the effects of FGFR2 on endometriosis progression.

## Conclusion

FGFR2 was demonstrated to be highly expressed in the ectopic endometrium of women with endometriosis. FGFR2 silencing in ESCs effectively repressed proliferation, migration, invasion, and EMT, as well as induced apoptosis. The ERK signaling pathway, in contrast to p38 and JNK signaling pathway, was shown to be a downstream signaling pathway for FGFR2 in the disease progression of endometriosis. These conclusions suggest that FGFR2 could be a potential therapeutic target for the treatment of endometriosis.

## Supplementary Material

Supplemental Material

## Data Availability

The datasets used and analyzed during the current study are available from the corresponding author on reasonable request.

## Highlights:

1. FGFR2 is highly expressed in endometriosis;
2. Silence of FGFR2 inhibits the growth of ectopic stromal cells;
3. Silence of FGFR2 inhibits the migration and invasion of ectopic stromal cells;
4. Silence of FGFR2 inhibits ERK signaling pathway in ectopic stromal cells;
5. FGFR2 promotes ectopic stromal cell proliferation and invasion via ERK signaling.
