# Supplementary material for: Fibroblast growth factor receptor 2 promotes the proliferation, migration, and invasion of ectopic stromal cells via activation of extracellular-signal-regulated kinase signaling pathway in endometriosis
Source: Bioengineered. 2022 Mar 21;13(4):8360–71. doi: 10.1080/21655979.2022.2054207 (PMC9161834; doi:10.1080/21655979.2022.2054207)
Supplement: Supplemental Material [file KBIE_A_2054207_SM2836.zip › supplementary/downloadFromZipFile.pdf]

伦理审查申请表

批准号：2020-065

研究基本信息				
项目名称	FGFR2 通过激活 ERK 信号通路促进异位基质细胞的增殖、迁移和侵袭			
项目类型	临床数据			
主要研究者	徐媛		承担科室	中医科
研究性质	□多中心（□组长单位 □参与单位/组长单位： ） √单中心			
研究周期	2020 年 2 月~2021 年 9 月			
研究者姓名	单位名称	专业背景	职称	负责事项
1. 主要研究者	徐媛	硕士	副主任医师	构思设计文章，获得实验数据
…				

研究具体内容			
研究目的	本研究旨在证明 FGFR2 在子宫内膜异位症中的作用。		
研究方法	□干预 □观察性（□前瞻性 □回顾性 □现况性） √样本采集/基础研究		
研究对象	□健康人 √患者（疾病： ）	样本量	
样本采集 类型	□血液 √组织 □其他：	是否出境	□是 √否
样本采集 来源	□生物样本库□既往留存 √计划采集	国际合作	□是 √否
使用的药物/器械/制剂名称		是否在国内上市	□是 √否
是否有前期有效的临床研究/基础研究/动物实验支持		□是 □否 √不涉及	
是否使用安慰剂		□是 □否 √不涉及	
是否有基础治疗		□是 □否 √不涉及	
✧ 无基础治疗且必须使用安慰剂的原因：			
是否涉及以下内容：			
弱势群体 √不涉及	□未成年人 □孕妇或胎儿 □晚期肿瘤/癌症患者 □精神障碍患者<br>□无阅读能力（文盲，视力障碍，智力障碍，意识障碍等） □高龄老人<br>□其他：		
	✧ 必须纳入的原因：		
知情同意	√书面知情同意（签署人：√本人□监护人□亲属）□免知情同意（须附申请）		
遗传学内容	√不涉及 □涉及（具体内容：）		
审查类别			

<table>
<tr><td colspan="4">√初始审查 □复审（□修正后同意 □重审） □修正案审查 □严重不良事件
□安全性报告审查 □方案违背审查 □进展报告审查 □提前终止/暂停研究审查
□结题审查 □其他</td></tr>
<tr><td colspan="4">送审文件清单</td></tr>
<tr><td colspan="4">☒临床研究方案（版本号： ，版本日期： ）</td></tr>
<tr><td colspan="4">☒知情同意书/免知情同意申请（版本号： ，版本日期： ）</td></tr>
<tr><td colspan="2">□主要研究者简历</td><td colspan="2">□受试者出现紧急医学不良事件应急预案（干预研究）</td></tr>
<tr><td colspan="2">□组长单位伦理批件（多中心非组长单位提供）</td><td colspan="2">□病历真实性证明（回顾性研究须提供）</td></tr>
<tr><td colspan="2">□研究者手册（版本号:，版本日期:）</td><td colspan="2">□病例报告表（版本号:，版本日期:）</td></tr>
<tr><td colspan="2">□招募受试者材料（版本号:，版本日期:）</td><td colspan="2">□试验药物、器械检验报告</td></tr>
<tr><td colspan="2">□药品、器械生产单位资质</td><td colspan="2">□药品、器械注册批件</td></tr>
<tr><td colspan="2">其他：</td><td colspan="2"></td></tr>
<tr><td>研究者承诺内容</td><td colspan="3">本人承诺待该项目批准后，我将遵循 GCP、方案以及伦理准则，开展本项临床研究。①及时上报研究过程中的各类信息，任何修订将事先报告伦理委员会，待批准后继续开展。在持续审查规定日期前一个月递交研究进展报告供伦理委员会审查，逾期未交报告而造成研究数据无法使用，由本人承担相应责任。③所有涉及人类遗传资源采集、收集、买卖、出口、出境的研究，待获得人类遗传办批件后再开展。</td></tr>
<tr><td>研究者利益冲突声明</td><td colspan="3">我作为本临床研究的主要研究者，在此研究中不存在经济上、物质上、以及社会关系方面的利益冲突。倘若在研究开展过程中发现目前尚未知晓的利益冲突，我将及时向伦理委员会报告。</td></tr>
<tr><td>主要研究者签字</td><td>徐媛</td><td>日期</td><td>2020年2月</td></tr>
</table>

送审说明：

1.《伦理审查申请表》单独双面打印；

2. 文件按“送审文件清单”顺序排列装订。
