# Supplementary material for: Fibroblast growth factor receptor 2 promotes the proliferation, migration, and invasion of ectopic stromal cells via activation of extracellular-signal-regulated kinase signaling pathway in endometriosis
Source: Bioengineered. 2022 Mar 21;13(4):8360–71. doi: 10.1080/21655979.2022.2054207 (PMC9161834; doi:10.1080/21655979.2022.2054207)
Supplement: Supplemental Material [file KBIE_A_2054207_SM2836.zip › supplementary/flow cytometry raw data.docx]

**Figure 2D si-NC group**


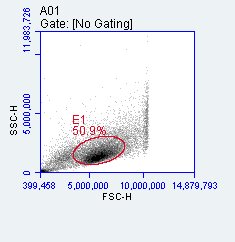

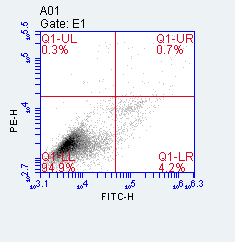


| **Plot 1: A01** | **Count** | **Events / μL** | **% of This Plot** | **% of All** | **Mean FSC-H** | **Mean SSC-H** | **CV FSC-H** | **CV SSC-H** | **Median FSC-H** | **Median SSC-H** |
| --- | --- | --- | --- | --- | --- | --- | --- | --- | --- | --- |
| All | 19,837 | 1653 | 100.00% | 100.00% | 4,381,003.94 | 1,328,033.36 | 69.24% | 89.27% |  |  |
| E1 | 10,102 | 842 | 50.93% | 50.93% | 5,884,640.96 | 1,515,043.29 | 16.21% | 31.67% |  |  |

| **Plot 2: A01: Gated on E1** | **Count** | **Events / μL** | **% of This Plot** | **% of All** | **Mean FITC-H** | **Mean PE-H** | **CV FITC-H** | **CV PE-H** | **Median FITC-H** | **Median PE-H** |
| --- | --- | --- | --- | --- | --- | --- | --- | --- | --- | --- |
| This Plot | 10,102 | 842 | 100.00% | 50.93% | 14,540.67 | 3,100.85 | 384.36% | 377.45% |  |  |
| Q1-UL | 23 | 2 | 0.23% | 0.12% | 18,738.48 | 65,233.30 | 31.61% | 89.66% |  |  |
| Q1-UR | 120 | 10 | 1.19% | 0.60% | 331,038.77 | 67,409.97 | 104.28% | 112.10% |  |  |
| Q1-LL | 9,459 | 788 | 93.63% | 47.68% | 6,667.70 | 1,953.59 | 62.87% | 46.00% |  |  |
| Q1-LR | 500 | 42 | 4.95% | 2.52% | 87,328.98 | 6,512.44 | 69.31% | 65.14% |  |  |

**Figure 2D si-1 group**


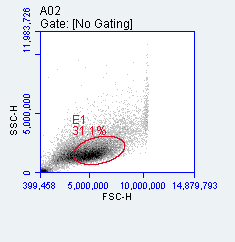

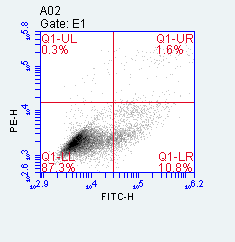


| **Plot 1: A02** | **Count** | **Events / μL** | **% of This Plot** | **% of All** | **Mean FSC-H** | **Mean SSC-H** | **CV FSC-H** | **CV SSC-H** | **Median FSC-H** | **Median SSC-H** |
| --- | --- | --- | --- | --- | --- | --- | --- | --- | --- | --- |
| All | 32,158 | 2474 | 100.00% | 100.00% | 2,624,192.34 | 994,565.97 | 103.52% | 119.22% |  |  |
| E1 | 10,000 | 769 | 31.10% | 31.10% | 5,285,797.33 | 1,667,355.36 | 19.76% | 28.06% |  |  |

| **Plot 2: A02: Gated on E1** | **Count** | **Events / μL** | **% of This Plot** | **% of All** | **Mean FITC-H** | **Mean PE-H** | **CV FITC-H** | **CV PE-H** | **Median FITC-H** | **Median PE-H** |
| --- | --- | --- | --- | --- | --- | --- | --- | --- | --- | --- |
| This Plot | 10,000 | 769 | 100.00% | 31.10% | 21,677.76 | 4,415.17 | 333.40% | 423.80% |  |  |
| Q1-UL | 30 | 2 | 0.30% | 0.09% | 13,391.37 | 64,680.73 | 53.30% | 82.59% |  |  |
| Q1-UR | 155 | 12 | 1.55% | 0.48% | 381,362.50 | 121,897.56 | 91.56% | 69.00% |  |  |
| Q1-LL | 8,733 | 672 | 87.33% | 27.16% | 7,365.64 | 2,058.53 | 72.73% | 48.93% |  |  |
| Q1-LR | 1,082 | 83 | 10.82% | 3.36% | 85,897.02 | 4,935.29 | 92.14% | 70.47% |  |  |

**Figure 2D si-2 group**


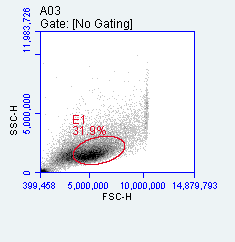

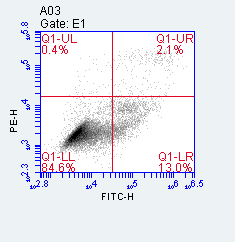


| **Plot 1: A03** | **Count** | **Events / μL** | **% of This Plot** | **% of All** | **Mean FSC-H** | **Mean SSC-H** | **CV FSC-H** | **CV SSC-H** | **Median FSC-H** | **Median SSC-H** |
| --- | --- | --- | --- | --- | --- | --- | --- | --- | --- | --- |
| All | 34,094 | 2435 | 100.00% | 100.00% | 2,728,633.01 | 1,032,287.01 | 101.35% | 117.66% |  |  |
| E1 | 10,885 | 778 | 31.93% | 31.93% | 5,288,413.39 | 1,658,308.09 | 19.76% | 28.62% |  |  |

| **Plot 2: A03: Gated on E1** | **Count** | **Events / μL** | **% of This Plot** | **% of All** | **Mean FITC-H** | **Mean PE-H** | **CV FITC-H** | **CV PE-H** | **Median FITC-H** | **Median PE-H** |
| --- | --- | --- | --- | --- | --- | --- | --- | --- | --- | --- |
| This Plot | 10,885 | 778 | 100.00% | 31.93% | 21,262.63 | 4,289.02 | 329.16% | 432.10% |  |  |
| Q1-UL | 35 | 3 | 0.32% | 0.10% | 10,764.09 | 32,080.09 | 56.69% | 88.57% |  |  |
| Q1-UR | 184 | 13 | 1.69% | 0.54% | 356,511.47 | 111,068.08 | 93.27% | 81.71% |  |  |
| Q1-LL | 9,514 | 680 | 87.40% | 27.91% | 7,329.59 | 2,071.57 | 71.57% | 50.91% |  |  |
| Q1-LR | 1,152 | 82 | 10.58% | 3.38% | 83,103.42 | 4,702.86 | 85.18% | 70.04% |  |  |
