# Supplementary material for: Fibroblast growth factor receptor 2 promotes the proliferation, migration, and invasion of ectopic stromal cells via activation of extracellular-signal-regulated kinase signaling pathway in endometriosis
Source: Bioengineered. 2022 Mar 21;13(4):8360–71. doi: 10.1080/21655979.2022.2054207 (PMC9161834; doi:10.1080/21655979.2022.2054207)
Supplement: Supplemental Material [file KBIE_A_2054207_SM2836.zip › supplementary/Original WB images.docx]

**Figure 1D**

FGFR2







β-actin







**Figure 2A**

FGFR2







β-actin







**Figure 3C**

E-cadherin







N-cadherin







Vimentin







β-actin







**Figure 4D**

p-ERK1/2







ERK1/2







p-p38







p38







p-JNK







JNK







β-actin







**Figure 5A**

FGFR2







p-ERK1/2







ERK1/2







β-actin
